# Ecosystem-engineered infections: beaver-modified wetlands are associated with conflicting drivers of amphibian pathogen prevalence

**DOI:** 10.1098/rsos.241169

**Published:** 2025-07-09

**Authors:** Leah M. Fischer, Angela D. Luis, Blake R. Hossack, Taegan A. McMahon, Winsor H. Lowe

**Affiliations:** ^1^ Wildlife Biology Program, W.A. Franke College of Forestry & Conservation, University of Montana, Missoula, MT 59812, USA; ^2^ Division of Biological Sciences, University of Montana, Missoula, MT 59812, USA; ^3^ US Geological Survey, Northern Rocky Mountain Science Center, Natural Sciences 113, University of Montana, Missoula, MT 59812, USA; ^4^ Department of Biology, University of Tampa, 410 West Kennedy Boulevard, Tampa, FL 33606, USA; ^5^ Department of Biology, Connecticut College, 270 Mohegan Avenue Parkway, New London, CT 06320, USA

**Keywords:** chytrid fungus, *Batrachochytrium dendrobatidis*, beaver, amphibian, chytridiomycosis, disease ecology

## Abstract

Beavers are ecosystem engineers and keystone species that protect freshwater resources and increase biodiversity. Beaver reintroductions are promoted for amphibian conservation, yet their impact on *Batrachochytrium dendrobatidis* (Bd), a pathogen linked with amphibian population declines worldwide, remains unclear. We investigated the abiotic and biotic drivers of Bd prevalence in Columbia spotted frogs (*Rana luteiventris*) and western toads (*Anaxyrus boreas*) in 20 beaver-modified and 23 non-beaver wetlands in Glacier National Park, USA. We found that beavers increased wetland hydroperiod, which was associated with higher Bd prevalence. However, beavers also reduced wetland canopy cover, which was associated with lower Bd prevalence. Our models also predicted higher Bd prevalence associated with higher adult density of both species of amphibians, although species’ densities were similar in beaver-modified and non-beaver wetlands. These results suggest that beavers have a cumulatively negligible net effect on Bd prevalence owing to their effects on both hydroperiod and canopy cover, which is encouraging for amphibian conservation. Our findings also suggest that decreasing canopy cover may be a potential management option to reduce Bd prevalence. In addition, these findings indicate that beaver-mimicking restoration projects may harm amphibian populations if they increase wetland hydroperiods without reducing canopy cover.

## 1. Introduction

During a time of unprecedented global biodiversity loss [1], beavers (*Castor* spp.) are increasingly used as a tool in conservation because they are ecosystem engineers that modify and maintain freshwater habitats and increase biodiversity [2–5]. Over the past century, North American beaver (*Castor canadensis*) populations have begun to rebound across the USA, recovering from their near-extirpation during the fur trade circa 1600−1900 [6]. By cutting down trees and building dams on streams, beavers create and maintain wetland habitats that are suitable for diverse communities of stream and pond-dwelling plant and animal taxa [2,7,8]. The value of these complex wetland habitats created by beavers has led to the promotion of beaver recoveries and reintroductions for the conservation of organisms that rely heavily on aquatic habitats for breeding and survival, including amphibians [9].

Beaver-modified wetlands provide a mosaic of habitat conditions suitable for a wide variety of amphibian species [10,11]. Amphibians are a taxon of particular conservation concern because they have experienced major declines globally over recent decades [12–14]. Most amphibians have complex life cycles that entail species-specific habitat requirements in both aquatic and terrestrial environments [15]. Wetland characteristics like hydroperiod (i.e. length of time that a wetland holds water), depth, wetland size, canopy cover and water flow are important for amphibian species to breed and survive [16,17]. Beavers create complex habitats with increased depth, larger area and longer hydroperiods than non-beaver habitats, thereby increasing local biodiversity of pond-breeding and pond-overwintering amphibian species [11,18,19]. Additionally, by felling trees and creating shallow flooded areas, beavers often increase water temperature in closed-canopy riparian systems and create thermal conditions favourable for a larger range of amphibian species [10,20].

In addition to these beneficial effects, beaver activity may influence interactions between amphibians and the parasites or pathogens that they encounter (hereafter referred to as ‘pathogens’) with potentially strong and wide-ranging consequences. Like their amphibian hosts, pathogens—especially those with free-living stages—often have species-specific abiotic ranges for survival and transmission [21,22]. These abiotic ranges lead to variations in pathogen occurrence and abundance across landscapes and even within wetlands [23,24]. Also, habitat modifications can influence the density of some amphibian host species, which can increase infections by pathogens that rely on high contact rates for transmission [25–29]. Consequently, habitat modifications can affect transmission by influencing pathogen survival in the environment and altering encounter rates of pathogens with susceptible amphibians [27,30,31].

*Batrachochytrium dendrobatidis* (Bd) is an amphibian pathogen that can be affected by habitat conditions and host species. Bd is a chytrid fungus that causes the disease chytridiomycosis, has spread to every continent where amphibians are found, and continues to cause amphibian population declines [32,33]. Bd is transmitted through direct contact between amphibians and through exposure to free-swimming, aquatic zoospores [32,34,35]. Bd prevalence (i.e. the proportion of infected amphibians in a population) can vary among wetlands owing to abiotic habitat characteristics, amphibian host susceptibility and host dynamics [33,36,37]. For example, increased water availability associated with permanent hydroperiods may increase Bd prevalence by preventing Bd zoospore desiccation [38–40]. Additionally, decreases in canopy cover resulting from cyclones and wildfires reduced Bd prevalence, probably by exposing Bd to drier conditions or higher temperatures ([24,41], but see [42]). Furthermore, the presence of specific hosts can increase infection prevalence in co-occurring species [43,44], and even the presence of tadpoles in ponds can increase Bd prevalence in adults [45]. High host density has also increased Bd prevalence ([46,47], but see [48]). Consequently, beaver-mediated habitat modifications to wetlands—by changing habitat characteristics and amphibian species characteristics important for Bd survival and transmission—could affect Bd prevalence.

Although Bd prevalence can be driven by environmental and host changes similar to those caused by beavers, the relationship between beaver-modified habitats and Bd prevalence has not been investigated directly. Surveys from 2002 to 2011 in national parks in the Rocky Mountains (USA) showed that amphibian occupancy was positively associated with beaver-modified wetlands, despite widespread presence of Bd [49]. Of the four parks included in that study, Glacier National Park had the highest proportion of beaver-modified wetlands, but long-term monitoring of western toads (*Anaxyrus boreas*) in Glacier National Park has shown that Bd is still reducing their survival in beaver-modified wetlands [50,51]. This suggests that although beaver-modified habitat may attract amphibians and even increase amphibian richness [11,18,49], it may also create conditions that are suitable for Bd survival and transmission, potentially reducing amphibian viability. Although these potential interactions between beavers and Bd prevalence are not yet understood, beaver-based restorations, including beaver transplanting and beaver reintroductions, are still being encouraged and implemented [4,9].

Here, we test the overarching hypothesis that beavers affect Bd prevalence within wetlands by modifying abiotic and biotic conditions that influence the performance and transmission of Bd. The objectives of this study were to identify key characteristics common to beaver-modified wetlands and determine how these characteristics influence Bd prevalence in amphibians. To test our hypothesis, we measured Bd prevalence in western toads and Columbia spotted frogs (*Rana luteiventris*) in beaver-modified and non-beaver wetlands in Glacier National Park. We predicted beavers would directly influence Bd prevalence by decreasing canopy cover or by increasing hydroperiod. If beavers were found to decrease canopy cover, then we expected lower Bd prevalence in amphibians in beaver-modified wetlands than non-beaver wetlands. If beavers increase hydroperiod, then we expected higher Bd prevalence in amphibians in beaver-modified wetlands than non-beaver wetlands. However, based on prior research showing both western toads and spotted frogs were more likely to inhabit beaver-modified wetlands [49], we predicted beavers would also increase Bd prevalence indirectly by increasing frog and toad densities. To evaluate these potential competing drivers of Bd prevalence, we used generalized linear models to assess the relationship among habitat characteristics, amphibian density and Bd prevalence in frogs and toads. By weighing the influence of each factor with model selection and examining the marginal effects of these variables, we aimed to determine which drivers exert the strongest influence on Bd infections. Our overarching goal was to provide novel insight into the value of beavers as a tool for amphibian conservation, especially during a time when beaver recolonization and reintroductions are being promoted in North America.

## 2. Methods

### 2.1. Study area and wetland selection

Glacier National Park is located in northwestern Montana, USA, and encompasses over 1000 wetlands that provide vital protected habitat for amphibians [49]. The park straddles the Continental Divide and is dominated by montane and subalpine forests spanning 975–3100 m in elevation. Beavers in this region were protected from over-exploitation before the establishment of Glacier National Park in 1910 by the Blackfeet Nation, and beaver populations have remained stable in the park since that time [52].

**Figure 1.**
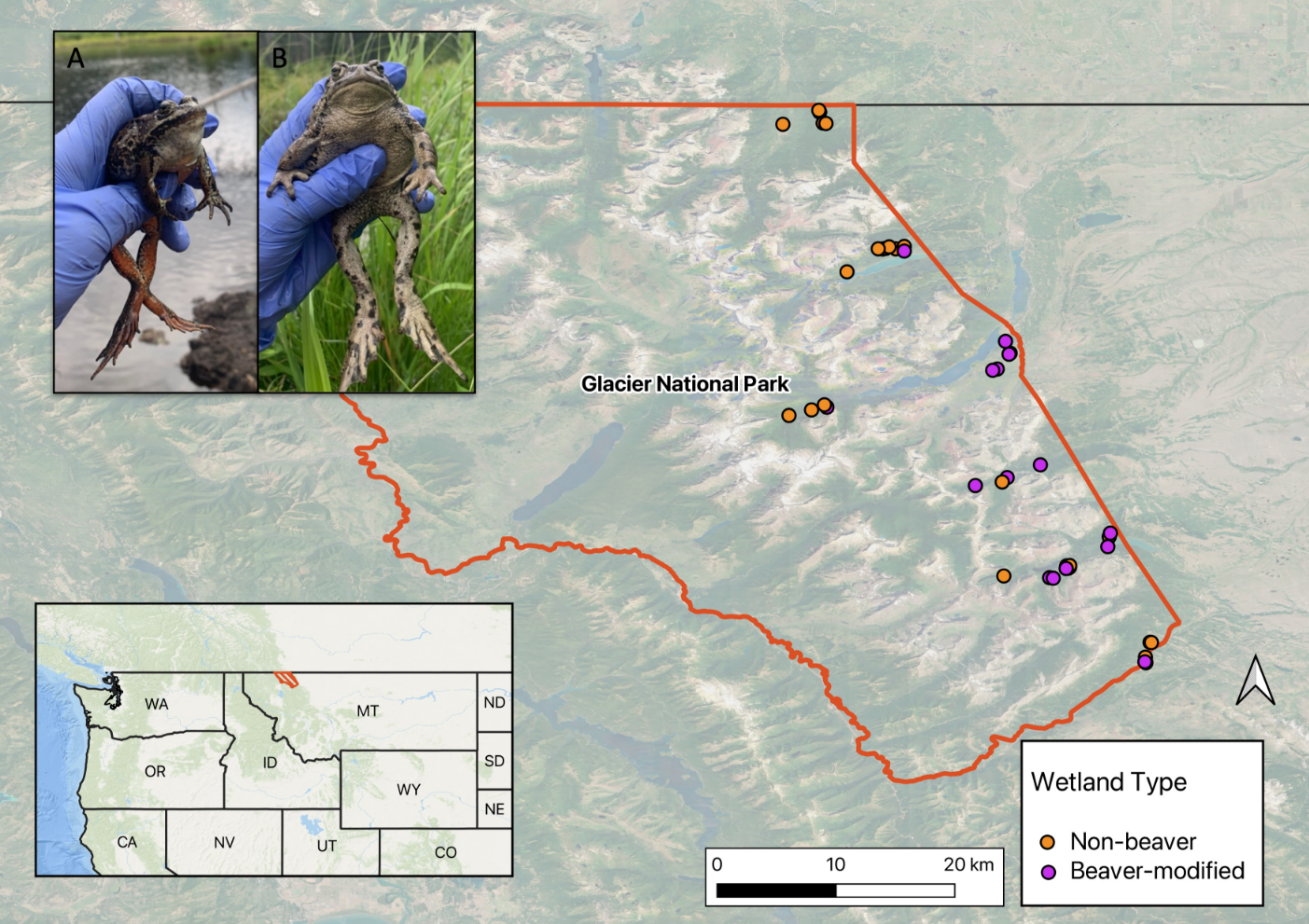
Locations of study wetlands in Glacier National Park in Montana, USA. The map inset shows where Glacier National Park is situated in northwest Montana. Purple points indicate beaver-modified wetlands (*n* = 20). Orange points indicate non-beaver wetlands (*n* = 23). The inset photos show a researcher holding a Columbia spotted frog (*a*) and a western toad (*b*).

Surveys of over 1000 wetlands throughout Glacier National Park from 2002 to 2011 provided a dataset we relied heavily on to select our study wetlands [49]. Specifically, we selected wetlands based on known amphibian breeding locations [49] and by visual inspection of maps of likely amphibian-occupied wetlands. We also selected wetlands distributed across major drainages within Glacier National Park and we prioritized wetlands east of the Continental Divide and in valley bottoms (1371−1817 m) where beaver activity is most common (figure 1). Beaver-modified and non-beaver wetlands share similar habitat features, such as varying amounts of canopy cover and both permanent and seasonal hydroperiods. We sampled comparable numbers of each wetland type within each major drainage to ensure a balanced representation of beaver-modified and non-beaver wetlands.

### 2.2. Study species

Of the six amphibian species documented in Glacier National Park, we focused on two of the most commonly occurring species: the western toad and the Columbia spotted frog (figure 1). Columbia spotted frogs are highly aquatic, whereas western toads are more terrestrial and tolerant to desiccation; however, both are found in similar wetlands throughout the spring and summer, including beaver-modified wetlands [53,54]. Columbia spotted frogs overwinter in aquatic habitats, and western toads migrate to upland habitats to hibernate underground in burrows [55]. Neither species can overwinter as tadpoles [53].

Both species are considered susceptible to Bd [50]. Population extirpations of western toads in the Rocky Mountains have been attributed to chytridiomycosis, but toads can behaviourally avoid or clear Bd infections by preferentially using microclimates unfavourable for Bd survival [24,50,56]. The long-toed salamander (*Ambystoma macrodactylum*) often breeds in the same wetlands where Columbia spotted frogs and western toads occur; however, Bd infections are rare in this species [24] and adults are largely terrestrial. We did not encounter adult salamanders at any of our study wetlands.

### 2.3. Wetland characteristics

At each wetland, we recorded elevation and walked the shoreline to calculate wetland surface area using Gaia GPS (https://www.gaiagps.com/) and a handheld Garmin eTrex 32 x. We established six habitat sampling locations around each wetland to measure physical and chemical conditions. Sampling locations were spaced equidistant around the perimeter of the wetland, and data were collected while standing in the water 1 m from the shore. At each sampling location, we measured water temperature, pH, salinity, total dissolved solids, conductivity and depth. We also estimated canopy cover with a convex spherical densiometer [57].

Wetlands were classified as beaver-modified if we documented signs of beaver presence signified by lodges or dams with beaver-chewed wood [49]. Hydroperiod was classified as seasonal (drying most years) or permanent (drying less than once every 10 years). If wetlands were not previously surveyed by Hossack *et al*. [49] and hydroperiod was unknown, we used historical imagery available from Google Earth Pro from 2004 to 2018 to assess whether the wetland had water in the years leading up to our surveys [58]. We also documented any water flowing into and out of each wetland during our surveys and characterized wetlands as either with or without flow. We used the programme PRISM [59] to estimate mean air temperatures and total precipitation at each wetland over the 30 days leading up to each survey to a 4 km resolution.

### 2.4. Amphibian sampling and density

Along with habitat assessments, we surveyed amphibians at each wetland twice within a one week period. During each survey, two surveyors walked the perimeter of the wetland for at least 30 min and captured all juveniles and adults (greater than 23 mm snout-to-vent length (SVL)) of both species. We captured all amphibians by hand while wearing disposable vinyl gloves and using established field protocol and sanitation methods to avoid spreading Bd [60,61]. All animals were stored individually in quart-sized bags (0.95-liter) until processing, which did not exceed 1 h. We recorded the sex, mass, SVL and life stage (juvenile or adult) for all individuals. Because tadpoles have been implicated as important Bd reservoirs [62], we used a dip net to sample 1 m of the shoreline every 3 m around the wetland perimeter to determine whether any tadpoles were present at wetlands.

To estimate density and to avoid sampling or swabbing the same individual more than once, we marked every captured adult or juvenile by clipping the tip of the rear right centre toe [63]. We estimated abundance and calculated standard errors by using the Chapman modification of the Lincoln–Petersen abundance estimator, using proportions of new and recaptured individuals across the surveys at each wetland [64,65]. We divided the abundance of Columbia spotted frogs and the abundance of western toads by wetland surface area to calculate densities at each wetland [66].

### 2.5. *Batrachochytrium dendrobatidis* sampling and analysis

To quantify Bd prevalence in Columbia spotted frogs and western toads, we swabbed up to 15 frogs and up to 15 toads at each wetland using established field protocol and sanitation methods [60]. Prevalence was calculated separately for frogs and toads as the proportion of positive individuals out of the total number of swabbed individuals. We used a standardized swabbing technique where we rubbed a sterile, rayon-tipped swab over the ventral surface, front legs, rear legs and feet of each amphibian 10 times to ensure equal sampling across wetlands and individuals [67]. Swabs were stored in sterile, screw-top 2 ml vials (Thermo Scientific, Waltham, MA, USA) and frozen until analysis. Swabs from 2018 were analysed at the University of Tampa, and swabs from 2019 were analysed at the University of Montana. The same DNA extraction and assay protocols were followed at both laboratory locations.

DNA was extracted from swabs using the 40 μl PrepMan Ultra Sample Preparation Reagent (Applied Biosystems, Foster City, CA, USA) protocol established by Hyatt *et al*. [68]. Real-time quantitative polymerase chain reaction (qPCR) assays for Bd detection and quantification were run in single wells on each swab following protocol by Boyle *et al*. [69], using TaqMan Exogenous Internal Positive Control Reagents (Applied Biosystems, Foster City, CA, USA) to detect inhibition and minimize Type I errors (false negatives) as outlined in Hyatt *et al*. [68]. Any swabs with inhibition were re-diluted and re-run. Swabs were considered positive for Bd if the qPCR reaction produced a clear sigmoidal amplification curve and a threshold cycle (Ct) value between 22 and 38. Lower Ct values indicate a higher initial quantity of Bd DNA, while values closer to 40 were deemed negative owing to potential nonspecific amplification [69].

### 2.6. Statistical analyses

We performed all statistical analyses in RStudio [70] and conducted analyses in two stages. First, to identify how abiotic and biotic characteristics differ in beaver-modified wetlands and non-beaver wetlands, we compared physical habitat characteristics, water quality indicators, frog and toad population densities and tadpole presence between wetland types. We then used generalized linear models and model selection methods to determine how habitat characteristics and amphibian population attributes were associated with Bd prevalence in frogs and toads. By weighing the influence of each factor through model selection and examining the marginal effects of these variables, we then compared these variables to determine which drivers exert the strongest influence on Bd prevalence. Prior to regression analyses, we tested for correlation between covariates to ensure variables in our models were independent.

### 2.7. Comparison of beaver-modified and non-beaver wetlands

To determine how abiotic and biotic characteristics of beaver-modified wetlands differ from non-beaver wetlands, we compared physical habitat characteristics (i.e. elevation, canopy cover, hydroperiod, flow, depth and surface area), water quality indicators (i.e. pH, salinity, conductivity) and amphibian population attributes (i.e. frog and toad densities and tadpole presence). We used Wilcoxon and Welch *t*-tests for continuous variables and Pearson’s chi-square tests for categorical variables (i.e. flow (1 = flow, 0 = no flow), hydroperiod (1 = permanent, 0 = seasonal) and tadpole presence (1 = present, 0 = absent) [71]). We square-root or log-transformed continuous data to conform to assumptions for statistical models. To account for multiple comparisons, we used a Bonferroni-corrected *p*‐value (0.0042) for the total number of variable assessments [72].

### 2.8. Variable correlations

To avoid using correlated predictor variables in our regression models of Bd prevalence in frogs and toads, we calculated pairwise Pearson correlation coefficients between all wetland-level variables. We used a cut-off correlation coefficient of *r* = 0.50. We then used two strategies to remove correlated variables from further analyses. We chose one variable as a proxy for correlated variables if we expected that variable to be more important in influencing Bd prevalence. If we had no *a priori* reason to expect one variable to be more important in influencing Bd prevalence, we used principal component analysis (PCA) to generate summary indices of multicollinear variables [73]. PCAs were conducted with the R function ‘prcomp’ [74].

### 2.9. Generalized linear models for *Batrachochytrium dendrobatidis* prevalence

To identify abiotic and biotic variables affecting Bd prevalence in frogs and toads, we used generalized linear models (package: ‘lme4’; family: binomial; function: glm) in R, standardizing all continuous variables between 0 and 1 (package: ‘metan’; function: resca) [75,76]. We used a binomial error distribution with a logit link function to represent the proportion of infected individuals, as Bd infection status is binary (infected or not infected). The analyses for Bd prevalence in toads and frogs were conducted separately. Prevalence was weighted by the total number of swabbed frogs or toads per wetland, which ranged from 1 to 15. Because beaver-modified wetlands share abiotic and biotic characteristics with non-beaver wetlands, we included these characteristics in our models (table 1) rather than using beavers as a separate predictor. Parsimonious models were selected using backwards step-wise model selection by second-order Akaike’s information criterion (AICc) and Akaike weights [71,77]. Backwards step-wise model selection starts with a model that includes all predictors and iteratively removes the least significant variable, based on a *p*-value of 0.05, until only those that contribute meaningfully to the model remain. The final model, therefore, includes only the most significant predictors [71]. Second-order AICc is a modified version of the AIC that includes an additional correction for small sample sizes, making it more accurate in model selection when the sample size is limited [78].

**Table 1.** Wilcoxon and Welch two-sample *t*-tests or Pearson’s chi-squared test results, degrees of freedom (*d.f.*), *p*-values, mean estimates (with standard deviation estimates in parentheses) of flow, permanent hydroperiod, tadpole presence, canopy cover, surface area, elevation, frog and toad density, pH, salinity, conductivity and depth between beaver-manipulated (*n* = 20) and non-beaver (*n* = 23) wetlands. (*p*-values in bold are significant according to the Bonferroni corrected *p*-value of 0.05/12 = 0.0042.)

variable	*t* or $\chi^{2}$	d.f.	*p*‐value	beaver mean (s.d.)	non-beaver mean (s.d.)
flow	$\chi^{2}$ *= 20.88*	1	**<0.0001**	19 out of 20 had flow	6 out of 23 had flow
hydroperiod	$\chi^{2}$ = 14.47	1	**0.0001**	20 out of 20 permanent hydroperiod	11 out of 23 permanent hydroperiod
tadpole presence	$\chi^{2}$ = 0.001	1	0.47	15 out of 19 had tadpoles	18 out of 23 had tadpoles
canopy cover	*t = 3.86*	40.6	**0.0003**	21 (18) %	43 (19) %
surface area	*t* = 2.40	2.51	0.02	4025 (3219) m^2^	1825 (2453) m^2^
elevation	*t* = 2.37	40.24	0.02	1539 (92) m	1617 (122) m
frog density	*t* = 1.67	40.98	0.10	0.10 (0.09) frogs m^−2^	0.15 (0.10) frogs m^−2^
toad density	*t* = 1.05	38.10	0.30	0.07 (0.07) toads m^−2^	0.05 (0.06) toads m^−2^
pH	*t* = −0.84	42.32	0.40	7.49 (0.69)	7.32 (0.67)
salinity	*t* = −0.70	36.31	0.49	0.15 (0.12) ppt	0.13 (0.11) ppt
conductivity	*t* = −0.68	36.59	0.49	293 (247) µS cm^−1^	258 (219) µS cm^−1^
*depth*	*t* = −0.69	40.81	0.49	20.5 (11.5) cm	18.0 (12.3) cm

Lastly, we generated *post hoc* predictions using estimates from our best models. We used the ‘margins’ package [79] to calculate marginal effects of the significant predictors on Bd prevalence in frogs and toads, paying particular attention to the predictors that are also correlated to beaver-modified habitat. This package calculates the marginal effect of a predictor variable with a 95% confidence interval by using estimated coefficients from a model while setting the other predictor variables to their mean values.

## 3. Results

### 3.1. Field surveys

We captured 890 Columbia spotted frogs and 451 western toads across 43 wetlands in June–August of 2018 and 2019 (figure 1). We classified 20 wetlands as beaver-modified wetlands and 23 wetlands as non-beaver wetlands (electronic supplementary material, table S1). We sampled 11 of these wetlands in 2018 (seven beaver-modified and three non-beaver) and 32 wetlands in 2019 (13 beaver-modified and 19 non-beaver). Frogs and toads occurred together in 27 wetlands, 13 wetlands had only Columbia spotted frogs and three had only western toads. Abundances of amphibians at each wetland were based on estimates from two capture sessions and were highly variable among all wetlands (electronic supplementary material, table S1). Abundance estimates ranged from 0 to 170 Columbia spotted frogs and 0 to 129 western toads (mean abundance ± s.d.; 29 ± 36 frogs; 12 ± 25 toads); densities ranged from 0 to 0.527 frogs m^−2^ and 0 to 0.263 toads m^−2^ (mean density ± s.d.; 0.161 ± 0.109 frogs m^−2^; 0.094 ± 0.063 toads m^−2^).

We detected Bd at every wetland. Specifically, 322 of 492 Columbia spotted frog swabs were Bd-positive, and 152 of 227 western toad swabs were Bd-positive. Prevalence for frogs and toads at each wetland ranged from 0 to 100% and was similar between species (Bd prevalence mean ± s.d.; frog Bd = 65 ± 25%; toad Bd = 62 ± 32%; *t*_53_= 0.35, *p* = 0.72; electronic supplementary material, table S1). Frog Bd prevalence was similar in beaver-modified and non-beaver wetlands (Bd prevalence mean ± s.d.; beaver-modified Bd = 62 ± 22%; non-beaver Bd = 67 ± 27%; *t*_37_= −0.55, *p* = 0.59). Toad Bd prevalence was similar in beaver-modified and non-beaver wetlands (Bd prevalence mean ± s.d.; beaver-modified Bd = 63 ± 27%; non-beaver Bd = 62 ± 38%; *t*_23_= 0.05, *p* = 0.96).

We sampled over Julian days 158–205 in 2018 and 148–214 in 2019. Mean monthly temperatures in the month leading up to sampling ranged from 8.8 to 13.7°C in 2018 and 4.9 to 15.2°C in 2019 (mean ± s.d.; 11.4 ± 1.7°C in 2018; 11.4 ± 2.8°C in 2019; electronic supplementary material, table S2). Total precipitation in the month leading up to sampling ranged from 20 to 111 mm in 2018 and 42 to 136 mm in 2019 (mean ± s.d.; 71 ± 31 mm in 2018; 83 ± 28 mm in 2019; electronic supplementary material, table S2).

### 3.2. Comparison of beaver-modified and non-beaver wetlands

**Figure 2.**
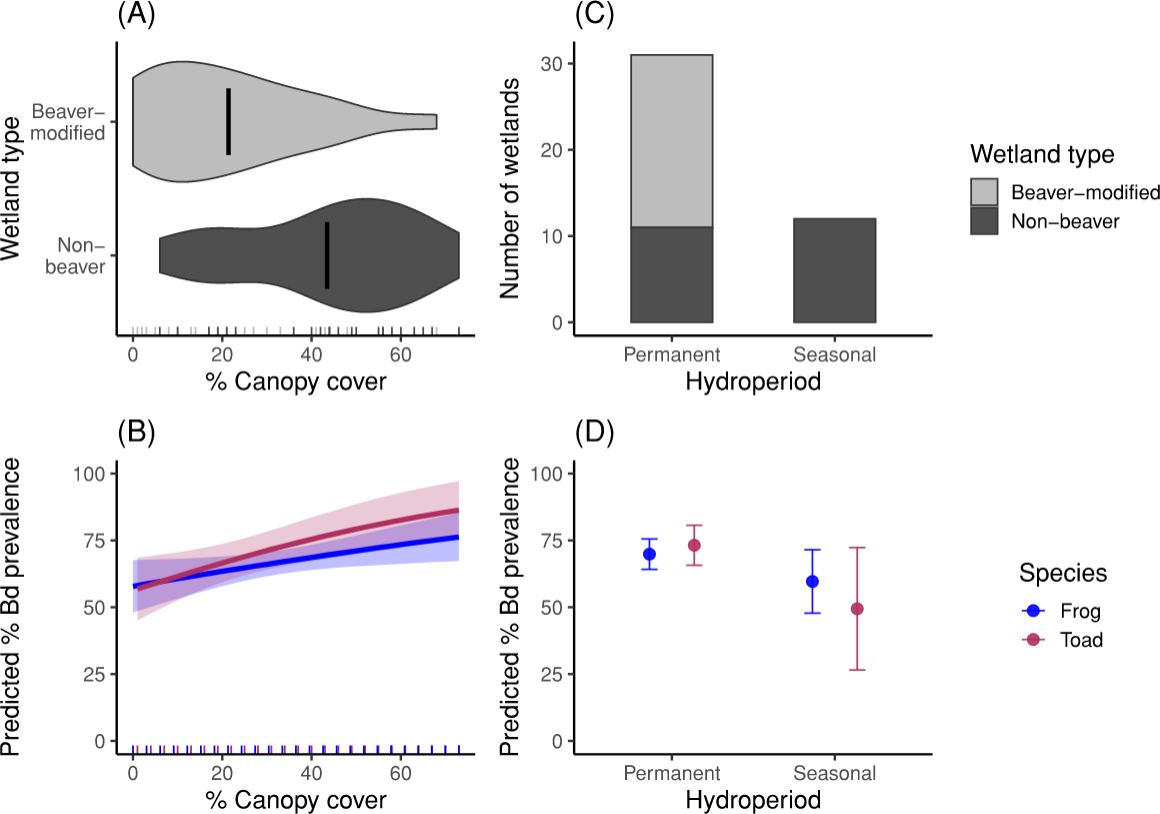
(A) Per cent canopy cover was significantly lower in beaver-modified wetlands than in non-beaver wetlands, and (B) areas with lower canopy cover had lower Bd prevalence based on the predicted marginal effects from best models for frogs and toads. Mean canopy cover measured in beaver-modified wetlands was 21% compared to 43% in non-beaver wetlands. (C) All beaver-modified wetlands (*n* = 23) had permanent hydroperiods compared to non-beaver wetlands (*n* = 23), which consisted of 12 permanent and 11 seasonal hydroperiods. (D) Columbia spotted frogs and western toads in wetlands with permanent hydroperiods had higher Bd prevalence based on the predicted marginal effects from the best model for toads and the second-best model for frogs. Shaded error bands and error bars represent 95% confidence intervals. Tick marks on the axes represent the distribution of those variables in the dataset.

Beaver-modified wetlands had less canopy cover than non-beaver wetlands (mean canopy cover ± s.d.: beaver-modified = 21 ± 18%; non-beaver = 43 ± 19%; *t* = 3.86, *p* = 0.0003; see figure 2A). Beaver-modified wetlands were also more likely to have permanent hydroperiods than non-beaver wetlands (*χ*^2^ = 14.47; *p* < 0.0001; see figure 2C) and more likely to have flowing water than non-beaver wetlands (*χ*^2^ = 20.88; *p* < 0.0001). Elevation, wetland surface area, pH, conductivity, salinity, depth at 1 m, toad density, frog density and tadpole presence did not differ significantly between beaver-modified and non-beaver wetlands (table 1; electronic supplementary material, table S3).

### 3.3. Variable correlation

Several physical and chemical variables were intercorrelated across wetlands. Flow was correlated with hydroperiod, canopy cover and wetland surface area (*r* = 0.62, −0.58 and 0.59, respectively). Surface area was correlated with hydroperiod (*r* = 0.64), and pH was correlated with salinity, total dissolved solids and conductivity (*r* = 0.61, 0.62 and 0.62, respectively). Julian day was correlated with mean daily air temperatures in the 30 days before sampling (*r* = 0.94) and total precipitation in the 30 days before sampling (*r* = 0.76), which were also correlated with one another (*r* = 0.79).

Because Julian day, mean temperatures and total precipitation are associated with climate seasonality and are known to influence Bd prevalence [80,81], we created a PCA to include these variables in linear models. The first principal component (climate PC1) explained 89.2% of the covariation, where lower values represented warm and dry climate conditions later in the summer and higher values represented cool and wet conditions earlier in the summer. Climate PC2 explained 8.7% of the covariation, where higher values represented wetter climate conditions later in the summer. We excluded climate PC3 from our models because it only explained 2.1% of the variation (electronic supplementary material, figure S1).

We included pH in models as a proxy for the other correlated water quality variables owing to its known influence on Bd prevalence and zoospore growth rates [82,83]. We included hydroperiod and canopy cover instead of flow in models for the same reason [40], and because we were not able to assess flow throughout the year (e.g. during peak discharge in the spring).

### 3.4. Generalized linear models for *Batrachochytrium dendrobatidis* prevalence

The stepwise-selected model for Bd prevalence in Columbia spotted frogs best supported by the data, as indicated by Akaike weights and AICc (table 2), included climate PC1, climate PC2, frog density and canopy cover. All parameters had positive coefficients (table 3), indicating that higher frog density and more canopy cover were associated with higher Bd prevalence and seasonality—specifically, cool and wet conditions early in the summer and wet conditions later in the summer—were associated with higher Bd prevalence. This model had a pseudo-*R^2^* of 0.31 and an Akaike weight of 0.47. Some support for hydroperiod was also included in the second-best model, which had an Akaike weight of 0.32 (table 2). Hydroperiod had a positive coefficient, indicating that permanent wetlands had higher predicted Bd prevalence than seasonal wetlands (table 4).

**Table 2.** Output of stepwise regression models of Bd prevalence in Columbia spotted frogs (A) and western toads (B) in Glacier National Park, Montana, USA, using abiotic covariates (i.e. permanent hydroperiod, canopy cover, elevation, pH and climate PCA axes PC1 and PC2) and biotic covariates (i.e. frog density, toad density and tadpole presence (‘tadpoles’)). (Second-order Akaike’s information criterion value differences (ΔAICc), AICc weights (AICc wt), number of estimable parameters (*K*) and deviances are listed for each model.)

(A) Bd prevalence in frogs				
model	ΔAICc	AICc wt	*K*	deviance
canopy cover+PC1+PC2+frog density	0.00	0.47	5	86.18
canopy cover+PC1+PC2+frog density+hydroperiod	0.79	0.32	6	84.19
*c*anopy cover+PC1+PC2+frog density+hydroperiod+ pH	2.53	0.13	7	82.98
canopy cover+PC1+PC2+frog density+hydroperiod+ pH+toad density	3.92	0.07	8	81.25
canopy cover+PC1+PC2+frog density+hydroperiod+ pH+toad density+tadpoles	6.94	0.01	9	80.89
canopy cover+PC1+PC2+frog density+hydroperiod+ pH+toad density+tadpoles+ elevation	10.49	<0.01	10	80.85

(B) Bd prevalence in toads				
model	ΔAICc	AICc wt	*K*	deviance
canopy cover+hydroperiod+ elevation	0.00	0.59	4	71.63
canopy cover+hydroperiod+ elevation+toad density	1.75	0.24	5	70.48
canopy cover+hydroperiod+ elevation+toad density+PC2	2.99	0.13	6	68.56
canopy cover+hydroperiod+ elevation+toad density+PC2+PC1	5.98	0.03	7	68.12
canopy cover+hydroperiod+ elevation+toad density+PC2+PC1+frog density	9.71	<0.01	8	68.08
canopy cover+hydroperiod+ elevation+toad density+PC2+PC1+frog density+pH	13.83	<0.01	9	68.06
canopy cover+hydroperiod+ elevation+toad density+PC2+PC1+frog density+pH+ tadpoles	18.40	<0.01	10	68.04

**Table 3.** Regression parameters, coefficient estimates, standard error (s.e.) and partial *p*-statistic for variables in the best models for Bd prevalence in Columbia spotted frogs (A) and Bd prevalence in western toads (B). (Frog density is denoted with an asterisk to indicate that it was square root transformed for the analysis.)

(A) Bd prevalence in frogs			
model parameter	estimate	s.e.	*p*
(intercept)	−0.990	0.288	<0.001
canopy cover	0.850	0.411	0.039
PC1	1.478	0.432	<0.001
PC2	0.727	0.471	0.123
frog density*	1.005	0.509	0.048

(B) Bd prevalence in toads			
model parameter	estimate	s.e.	*p*
(intercept)	−2.146	0.783	0.006
canopy cover	1.566	0.619	0.011
permanent hydroperiod	1.027	0.546	0.060
elevation	3.389	0.792	<0.001

**Table 4.** Regression parameters, coefficient estimates, standard error (s.e.) and partial *p*-statistic for variables in the second-best frog (A) and toad (B) Bd prevalence models. (Toad and frog density are denoted with an asterisk to indicate that they were square root transformed for the analysis.)

(A) frog Bd prevalence			
model parameter	estimate	s.e.	*p*
(intercept)	−1.608	0.535	0.003
canopy cover	1.030	0.432	0.017
PC1	1.455	0.429	<0.001
PC2	0.895	0.491	0.068
frog density*	1.481	0.619	0.017
*permanent hydroperiod*	0.451	0.321	0.161

(B) toad Bd prevalence			
model parameter	estimate	s.e.	*p*
(intercept)	−2.389	0.808	0.003
canopy cover	1.546	0.613	0.012
permanent hydroperiod	1.111	0.545	0.042
elevation	3.195	0.799	<0.001
toad density*	0.672	0.636	0.291

The stepwise-selected model for Bd prevalence in western toads best supported by the data included canopy cover, hydroperiod and elevation (table 2). This model had a pseudo-*R^2^* of 0.30 and an Akaike weight of 0.59. All parameters had positive coefficients (table 3), indicating that permanent hydroperiods had higher predicted Bd prevalence than seasonal hydroperiods, and more canopy cover and higher elevations were associated with higher Bd prevalence. Some support for toad density was also included in the second-best model, which had an Akaike weight of 0.24 (table 2). Toad density had a positive coefficient, indicating that higher toad densities were associated with higher Bd prevalence (table 4).

### 3.5. *Post hoc* predictions of beaver impacts on *Batrachochytrium dendrobatidis* prevalence

Our models indicated opposing influences of beavers on Bd prevalence; beavers decreased canopy cover (figure 2A) which decreases Bd (figure 2B), while they also increased hydroperiod (figure 2C) which increases Bd (figure 2D). To better understand these opposing influences of beavers on Bd, we calculated and plotted the marginal effects of these predictors on Bd prevalence in frogs and toads.

First, we calculated the marginal effects of canopy cover on Bd prevalence in frogs and toads, using their respective best models (table 3). We found that as canopy cover increased from 0 to 73% (the range observed), Bd prevalence in frogs (figure 2B, blue) was predicted to increase from 58 to 76%, and Bd prevalence in toads (figure 2B, red) was predicted to increase from 57 to 86%. The average beaver-modified wetland had a canopy cover of 21%, and this marginal effect is predicted to lead to 64% Bd prevalence in frogs and 67% Bd prevalence in toads. Non-beaver wetlands had an average canopy cover of 43%, which is predicted to lead to a Bd prevalence of 70% Bd prevalence in frogs and 76% Bd prevalence in toads.

Next, we calculated the marginal effects of hydroperiod on Bd prevalence in frogs and toads. For frogs, we used estimates from our second-best model (table 4). For toads, we again used estimates from our best toad model (table 3). We found that Bd prevalence in frogs (figure 2D, blue) is predicted to be 69% in wetlands with permanent hydroperiod compared to 59% in wetlands with seasonal hydroperiod. Bd prevalence in toads (figure 2D, red) is predicted to be 73% in wetlands with permanent hydroperiods and only 49% in wetlands with seasonal hydroperiods.

**Figure 3.**
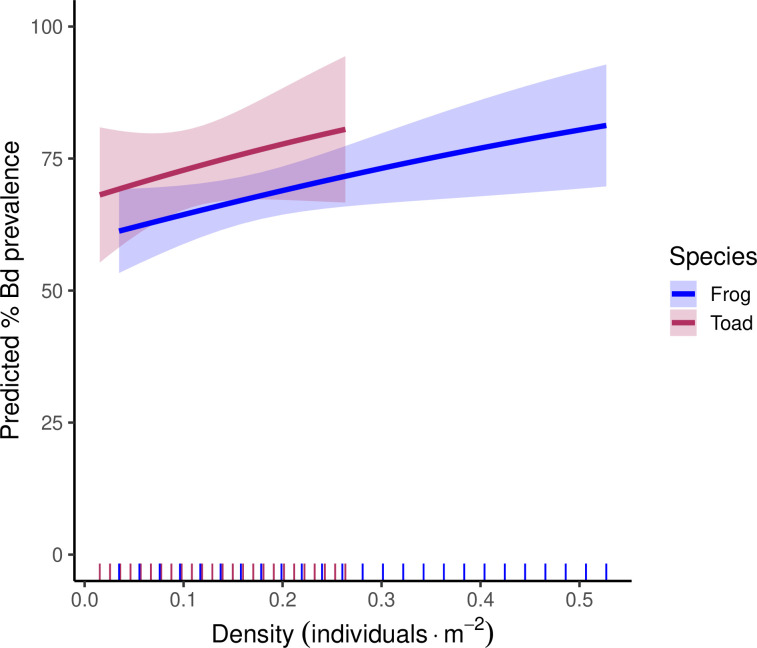
Marginal effects of host density on Bd prevalence predicted by the best model for Columbia spotted frogs and the second-best model for western toads. Hash marks along the bottom represent the distributions of density data for frogs (blue) and toads (red).

Our models also indicated that frog and toad densities were positively associated with Bd prevalence in both species. To predict the effect of densities on Bd prevalence, we plotted the marginal effects of density on Bd prevalence in frogs (figure 3, blue) and toads (figure 3, red). Frog density ranged from 0 to 0.53 frogs m^−2^ in our study. Over this range, Bd prevalence is predicted to increase from 60 to 81%. To predict Bd prevalence in toads, we used estimates from our second-best toad model (table 4). Toad density ranged from 0 to 0.26 toads m^−2^. Over this range, Bd prevalence is predicted to increase from 68 to 81%. Further exploration of combined marginal effects of the predictors in the best frog and toad models are presented in the electronic supplementary material.

## 4. Discussion

Our results show that habitat modifications associated with beavers influence Bd prevalence in opposing directions. Beaver-modified wetlands had less canopy cover, which was associated with decreased Bd prevalence in Columbia spotted frogs and western toads. Beaver-modified wetlands also had longer hydroperiods than non-beaver wetlands, which correlated to increased Bd prevalence in western toads, with a similar trend for increased Bd prevalence in Columbia spotted frogs. The effects of these two habitat characteristics are consistent with prior studies showing decreased Bd prevalence associated with less canopy cover [40,41,84,85] and increased Bd prevalence at wetlands with permanent hydroperiods [40,86]; however our study is, to our knowledge, the first to directly link these drivers of Bd prevalence to beaver-modified wetlands.

Bd transmission is considered density-dependent [47,62,87] and our findings further support this, particularly for frogs. Frog density was a top predictor for Bd prevalence in frogs, while there was some support for toad density being important for Bd prevalence in toads. Beaver-modified wetlands supported larger abundances of amphibians than non-beaver wetlands, but had larger surface areas than non-beaver wetlands, resulting in similar densities of frogs and toads between wetland types (table 1). Notably, beaver-modified wetlands that can support larger amphibian populations without increasing density may help maintain amphibian populations even in the presence of a lethal pathogen. This is further reinforced by our marginal effects plots, which predicted slightly higher Bd prevalence in non-beaver wetlands with permanent hydroperiods than in both seasonal non-beaver wetlands and beaver-modified wetlands with permanent hydroperiods (electronic supplementary material, figure S2C,D). This trend was significant only for frogs, but it underscores the importance of considering both density and the habitat characteristics associated with beavers (i.e. hydroperiod and canopy cover) in influencing pathogen prevalence. The drivers of Bd prevalence and the strength of their effects varied between amphibian species, which underscores the need for extended monitoring of additional amphibian species, ideally before and after beaver reintroductions, to quantify the impacts of beavers on Bd prevalence comprehensively.

Unrelated to beavers, yet consistent with literature, we found evidence that climate variables and elevation were important predictors of Bd prevalence in frogs and toads, respectively. Climate variables based on the loadings of our principal components (i.e. climate PC1 and PC2; electronic supplementary material, figure S1) were top predictors for Bd prevalence in frogs, with an increase in Bd prevalence observed in cooler and wetter conditions earlier in the summer, and in wetter conditions later in the summer. These trends align with previous studies linking wetter conditions to increased Bd prevalence [81,88,89]. However, we were surprised that climate variables were more important for Bd prevalence in frogs than in toads because of differences in habitat use by these two species. Given the more terrestrial nature of western toads compared to Columbia spotted frogs [24], we expected Bd infections in toads to be more dependent on climate-related variables (i.e. precipitation and temperature) than hydrological variables (i.e. hydroperiod and pH). Notably, Bd prevalence in toads increased with elevation, which is consistent with previous observations of Bd in Glacier National Park [90,91] and often attributed to seasonality and snowpack [92]. However, elevation in our study did not correlate with other climate variables, possibly owing to a narrower elevation range in our study compared to prior studies.

Our study highlights several important considerations for managing Bd and planning restoration projects. First, our findings suggest that reducing canopy cover could serve as a management strategy to mitigate the impact of Bd on amphibians. While previous studies have proposed canopy thinning to reduce Bd prevalence [40,85,93], to our knowledge, its implementation remains unexplored. When we investigated the marginal effects of canopy cover on Bd prevalence, we predicted that decreasing canopy cover from non-beaver wetlands (43%) to amounts measured surrounding beaver-modified wetlands (21%), could reduce Bd prevalence by 6% in frogs and by 9% in toads (figure 2B). Furthermore, Bd prevalence was predicted to be higher for both frogs and toads in wetlands with permanent hydroperiods compared to seasonal hydroperiods according to our marginal effects plots (figure 2D). After further investigation of the combined marginal effects of canopy cover and hydroperiod (electronic supplementary material, figure S2), we found that decreasing canopy cover was still effective at reducing Bd prevalence in both permanent and seasonal wetlands (electronic supplementary material, figure S2A,B). A previous study using data from Glacier National Park estimated a 35% reduction in mean annual survival when a toad was infected with Bd compared to when it was uninfected [51]. Reducing the prevalence of this pathogen through reductions in canopy cover may, therefore, serve as a protective measure against population declines.

Our results also indicate that restoration projects may pose a risk to amphibians if they only alter beaver-associated habitat conditions that increase Bd prevalence. Beaver dam analogues and other beaver mimicry restorations involving installation of dams along streams are being implemented across the western USA to mimic the ecological benefits of beavers [5,94–96]. However, in active beaver wetlands, beavers maintain dams that often sustain permanent hydroperiods, but they also continually reduce canopy cover surrounding the wetland over time [97]. Restorations involving beaver dam analogues or beaver mimicry should therefore be approached cautiously because, as our results show, solely increasing hydroperiods without thinning canopy cover could increase Bd prevalence. Comprehensive strategies that address both hydroperiod and canopy dynamics are essential for effective amphibian conservation in the context of Bd.

## Data Availability

All data and R code are provided in a FigShare repository (https://figshare.com/s/63654033226ace519e84). Only previously published R packages were used for this study's analysis and no new packages were developed. Supplementary material is available online [98].
